# The contribution of bile acid metabolism to the pathogenesis of *Clostridioides difficile* infection

**DOI:** 10.1177/17562848211017725

**Published:** 2021-05-28

**Authors:** Benjamin H. Mullish, Jessica R. Allegretti

**Affiliations:** Division of Digestive Diseases, Department of Metabolism, Digestion and Reproduction, Faculty of Medicine, Imperial College London, London, UK; Division of Gastroenterology, Hepatology and Endoscopy, Brigham and Women’s Hospital, 850 Boylston Street, Suite 201, Chestnut Hill, MA 02467, USA; Harvard Medical School, Harvard University, Boston, Massachusetts, USA

**Keywords:** bile acids, *Clostridioides difficile* infection, faecal microbiota transplant, farnesoid X receptor, gut microbiome, metabolomics

## Abstract

*Clostridioides difficile* infection (CDI) remains a major global cause of gastrointestinal infection, with significant associated morbidity, mortality and impact upon healthcare system resources. Recent antibiotic use is a key risk factor for the condition, with the marked antibiotic-mediated perturbations in gut microbiome diversity and composition that underpin the pathogenesis of CDI being well-recognised. However, only relatively recently has further insight been gained into the specific mechanistic links between these gut microbiome changes and CDI, with alteration of gut microbial metabolites – in particular, bile acid metabolism – being a particular area of focus. A variety of *in vitro, ex vivo*, animal model and human studies have now demonstrated that loss of gut microbiome members with bile-metabolising capacity (including bile salt hydrolases, and 7-α-dehydroxylase) – with a resulting alteration of the gut bile acid milieu *–* contributes significantly to the disease process in CDI. More specifically, this microbiome disruption results in the enrichment of primary conjugated bile acids (including taurocholic acid, which promotes the germination of *C. difficile* spores) and loss of secondary bile acids (which inhibit the growth of *C. difficile*, and may bind to and limit activity of toxins produced by *C. difficile*). These bile acid changes are also associated with reduced activity of the farnesoid X receptor pathway, which may exacerbate *C. difficile* colitis throughout its impact upon gut barrier function and host immune/inflammatory response. Furthermore, a key mechanism of efficacy of faecal microbiota transplant (FMT) in treating recurrent CDI has been shown to be restoration of gut microbiome bile metabolising functionality; ensuring the presence of this functionality among defined microbial communities (and other ‘next generation’ FMT products) designed to treat CDI may be critical to their success.

## Introduction

The burden of *Clostridioides difficile* infection (CDI) to global healthcare systems remains significant, with >460,000 cases occurring in the United States (US) alone in 2017.^1^ The condition remains the predominant cause of nosocomial gastrointestinal infection,^2^ although at least 25% of cases may be community acquired.^3,4^ Severe CDI is associated with an infection-related mortality of approximately 5%, and an all-cause mortality as high as 20%.^5^ The occurrence of CDI is recognised to quadruple the cost of hospitalisation, and is responsible for approximately US $1.5 billion of annual health expenditure in the US.^6^

It is well-established that recent antibiotic exposure (in particular, broad spectrum antibiotic use) is one of the major risk factors for the occurrence of CDI.^7^ Other associated risk factors include older age (>65 years),^3^ immunocompromise,^8^ use of proton pump inhibitors and enteral feeding.^9,10,11^ Antibiotics in particular – but all of these risk factors to at least some degree – are recognised to impact markedly upon the composition and functionality of the gut microbiome, with loss of key taxonomic features and reduction in microbial diversity being central contributors to the pathogenesis of the condition.^12^ More specifically, such disruption of the gut microbiome facilitates the colonisation of *C. difficile* in the distal gut, its germination, and its ability to undergo growth and toxin production.^12^ By extension, the central rationale for the use of faecal microbiota transplant (FMT) for the treatment of recurrent/refractory CDI is that it restores this damaged gut microbiome back towards a composition more resembling the pre-morbid situation.^13^

Advances in next-generation microbial sequencing (such as from stool or tissue samples of patients affected by CDI) have revealed ever-increasing granularity of detail as to the specific compositional changes to the gut microbiome occurring within the condition. However, such studies in themselves give only limited insight into the specific perturbances of gut microbiome functionality – and their impact upon host-microbiome crosstalk – that underlie CDI pathogenesis. Gut microbial-derived metabolites are recognised as one of the key intermediaries for interaction between the microbiome and the host, with their impacts on host physiology recognised as being as diverse as immune function and energy homeostasis.^14,15^ Bile acid metabolism is one major such function of the gut microbiome^16,17^ ; the recognition for over 20 years, initially from *in vitro* studies, that different bile acids have markedly different effects on the ability of *C. difficile* to undergo germination and vegetative growth was among the earliest clues of the importance of bile acids to the pathogenesis of CDI.

In this review, we present an overview of current knowledge regarding the contribution of disordered bile acid metabolism to the pathogenesis of CDI. We extend this further by exploring how current treatment modalities for CDI impact on bile acid metabolism, and potential future implications of this for novel CDI therapeutics.

## Overview of gut microbiome–bile acid interactions, and their relevance to CDI

**Figure 1.**
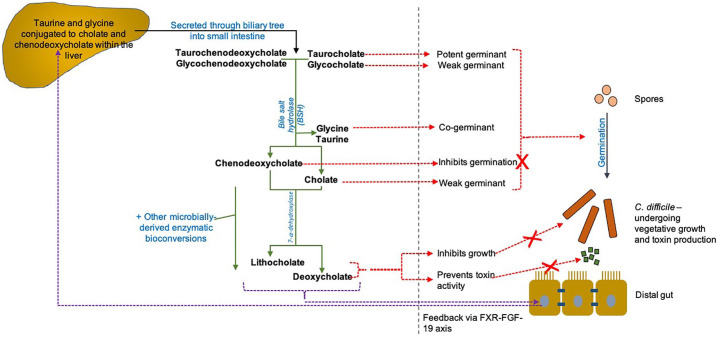
Schematic of gut microbiota–bile acid interactions in humans. The primary bile acids CA and CDCA are conjugated with taurine and glycine within the liver, and secreted through the biliary system into the small intestine. Once entering the distal gut, the enzyme BSH (found distributed widely amongst gut microbiome members) removes these taurine and glycine conjugates, reforming unconjugated CA and CDCA. Following on from this, the complex, multi-step process of 7-α-dehydroxylation also occurs through microbially derived enzymes, and converts primary to secondary bile acids (specifically, CA is converted to DCA, and CDCA is converted to LCA). A range of other microbially derived enzymes are also able to perform biotransformations upon primary bile acids, for example, 7 α/β-epimerisation to form ursodeoxycholic acid.^16^ TCA is the major endogenous trigger to *Clostridioides difficile* germination (with glycine as co-germinant); CA and DCA (at high concentrations) also trigger *C. difficile* germination.^22^ However, DCA and LCA at physiological concentrations inhibit TCA-mediated *C. difficile* germination^23,24^; these and other secondary bile acids also inhibit the vegetative growth and toxin activity of *C. difficile.*^22–25^ CDCA also inhibits the germination of *C. difficile*.^26^ The altered bile acid milieu found in the CDI gut is also associated with reduced signalling *via* the FXR-FGF-19 pathway, which may exacerbate CDI *via* a number of routes (see main text). Green arrows, microbially mediated biotransformations; red dotted arrows, impacts of bile acids upon the life cycle of *C. difficile*; purple dotted arrows, impacts of bile acids upon the FXR pathway. Adapted from Mullish *et al*.^27^ BSH, bile salt hydrolase; CA, cholate, CDCA, chenodeoxycholate, DCA, deoxycholic acid; FGF, fibroblast growth factor; FXR, farnesoid X receptor; LCA, lithocholic acid; TCA, taurocholate.

In humans and other mammals, bile acid production is a function of the liver, where conjugation of bile acids to the amino acids glycine and taurine also occurs. After secretion within bile from the liver (and concentration within the gallbladder), bile enters the small intestine. At this stage, a large proportion of the pool of bile acids are tauro- and glyco-conjugated versions of the group named ‘primary bile acids’, including cholic acid (CA) and chenodeoxycholic (CDCA).^18^ Biotransformation of primary bile acids to the group named ‘secondary bile acids’ [including deoxycholic acid (DCA) and lithocholic acid (LCA)] occurs within the distal small intestine and colon; this is undertaken by several enzymes produced by the gut microbiota, but not by mammals (Figure 1). The two key enzymatic steps of this process are a first step mediated by bile salt hydrolase (BSH; also known as choloylglycine hydrolase), which hydrolyses the glycine or taurine group from conjugated bile acids, and a subsequent step by 7-α-dehydroxylase, which converts unconjugated primary bile acids into secondary bile acids.^18,19^ While BSHs are found predominantly within the bacterial phyla *Bacteroidetes, Firmicutes* and *Actinobacteria*, they are distributed widely throughout most major bacterial divisions and archaea species of the gut microbiota.^19^ Furthermore, annotation for a *bsh* gene has been identified in 26.03% of all bacterial strains in the Human Microbiota Project microbiota reference genome.^20^ In contrast, based on current microbial genomic annotation, only a very small percentage of commensal gut microbiota members are predicted to possess 7-α-dehydroxylation activity, with those organisms that do predominantly belonging to the genera *Clostridium* clusters XIVa and XI.^18,21^ The biosynthesis of secondary bile acids creates a more hydrophobic bile acid pool, facilitating the elimination of such bile acids within faeces.

The potential relevance of bile acid metabolism to *C. difficile* infection was first suggested through the *in vitro* demonstration that different bile acids (and, in particular, primary *versus* secondary bile acids) had markedly different impacts upon different aspects of the ability of *C. difficile* to undergo different aspects of its life cycle. In particular, the conjugated primary bile taurocholic acid (TCA) potently promotes the germination of *C. difficile* (with glycine functioning as pro-germinant).^22,28^ The *C. difficile* bile acid germinant receptor was only relatively recently identified as CspC, a germination-specific protease.^29^ Unconjugated primary bile acids have differential effects on *C. difficile*, with CA mildly triggering germination,^22^ while CDCA inhibits this.^26^ The ability of CDCA to inhibit *C. difficile* germination is apparently more potent than CA in inducing it^22,26^; however, CDCA is absorbed by the colonic epithelium ten times faster than CA, therefore likely reducing levels below that needed for significant inhibition *in vivo*.^30^ Conversely, secondary bile acids (including both DCA and LCA) potently inhibit the vegetative growth and toxin activity of *C. difficile*^22,31^; in addition, DCA and LCA (at physiological concentrations) are able to inhibit TCA-mediated *C. difficile* germination.^23,31^ Another recent surprising finding has been that bile acids are able to directly bind to and inhibit the *C. difficile* TcdB toxin, with the secondary bile acids evaluated having more potent toxin binding capacity than their corresponding primary bile acids.^25^

## Experimental data regarding altered gut microbiome–bile acid interactions in CDI

Since bile acid metabolism is influenced so strongly by gut microbiota functionality – and given the marked effect of various bile acids upon different aspects of the *C. difficile* life cycle, as outlined above – it has been suspected that this pathway may also be relevant to the pathogenesis of CDI *in vivo*. A theory that has been investigated experimentally recently has been whether a key contributory mechanism to CDI pathogenesis is particularly antibiotic-related loss of gut microbial community members with BSH and/or 7-α-dehydroxylase functionality, with associated gut enrichment of TCA (promoting *C. difficile* germination) and loss of secondary bile acids (permitting vegetative growth and/or toxin activity of *C. difficile*) promoting progression of CDI.

Certain experimental work to date has focussed more towards the possible contribution of BSH to CDI. Allegretti and colleagues compared the stool microbiota of patients with a primary episode of CDI, patients with recurrent CDI and healthy controls.^32^ Using an inferential metagenomic tool (PICRUSt), researchers demonstrated that the predicted *bsh* gene abundance in the stool microbiota of rCDI patients was significantly lower than both patients with primary CDI and healthy controls. There was a trend observed towards reduced predicted stool *bsh* gene abundance in patients with primary CDI compared with controls, although this did not reach statistical significance. Using a random forest regression model, researchers were able to distinguish rCDI from primary CDI patients with an AUROC of ~0.84 using faecal bile acid profiling, with the ratio of DCA to glycoursodeoxycholic acid + DCA being the most important predictor variable for the model. Of note, this ratio was derived empirically, and there is no current experimental evidence evaluating the impact of glycoursodeoxycholic acid upon the life cycle of *C. difficile* directly.

Other experimental work has demonstrated the contribution of 7-α-dehydroxylase. Buffie and colleagues first applied mathematical modelling to compare gut microbiota profiles from antibiotic-exposed mice with different levels of CDI vulnerability, as well as to compare profiles from CDI patients with those who were *C. difficile* carriers.^33^ Using microbial sequencing, it was identified that the presence of an operational taxonomic unit (OTU) corresponding to *Clostridium scindens* was a key microbiota factor that predicted protection against CDI. In bacterial transfer experiments, researchers demonstrated that the degree of colonisation of the gut microbiota of antibiotic-treated mice with *C. scindens* correlated with degree of infection resistance. Since *C. scindens* is one of the few commensal bacteria with 7-α-dehydroxylase activity, the researchers hypothesised that the ability to produce secondary bile acids was the link between *C. scindens* colonisation and infection resistance. Evidence for this was reinforced by the demonstration that infection resistance was reversed after addition of the bile acid sequestrant cholestyramine (with cholestyramine being used in this experiment to bind and remove secondary bile acids).^33^ The same association between *C. scindens* and CDI resistance was observed after the organism was administered to germ-free mice.^34^ Of particular interest, a further potential role for 7-α-dehydroxylating bacteria in inhibiting the growth of *C. difficile* (in addition to secondary bile acid production) has also recently been identified. Specifically, *C. scindens* was identified *in vitro* as producing the tryptophan-derived antibiotic 1-acetyl-β-carboline, which inhibited the growth of *C. difficile*. This antibiotic is produced particularly in the presence of CA, and acts most potently against *C. difficile* in the presence of DCA or LCA.^35^

Further studies have demonstrated that the actions of both BSH and of 7-α-dehydroxylase together are likely to be contributory to CDI pathogenesis. In a study currently reported in abstract form only, 29 patients with primary CDI were monitored prospectively, and stool samples were collected once to twice weekly from diagnosis up until approximately 6 weeks if no recurrence occurred (*n* = 19) or until the point of recurrence (*n* = 10).^36^ Stool 16S rRNA gene sequencing was performed for each sample, and analysed using a machine-learning based approach; this demonstrated a greatly reduced rate of CDI recurrence occurring among patients whose gut microbiome contained *C. scindens* or *Clostridium hylemonae*, both 7-α-dehydroxylase-producing organisms. Further analysis demonstrated that, whereas levels of stool TCA significantly reduced over time (and levels of BSH activity restored) compared with baseline among patients who did not experience recurrence, neither changed significantly from baseline level in patients who went on to develop CDI recurrence.

## Impact of CDI treatments upon gut microbiome–bile acid interactions

### Antimicrobials

The current mainstays of antimicrobial therapy for CDI are vancomycin and fidaxomicin (with metronidazole having a much less prominent role in current treatment algorithms than those previously existing).^37^ Despite vancomycin and fidaxomicin both having established efficacy in reducing further recurrences when used in the treatment of rCDI, a proportion of patients will go on to have recurrences despite the use of these agents, and alternative treatment strategies are needed. While FMT is one key such strategy (and discussed further below), and further area of keen interest relates to novel antimicrobial therapies.

One such new drug of interest is ridinilazole, which has shown promising phase II trial results in the treatment of CDI, and is now awaiting phase III studies.^38,39^ Ridinilazole is bactericidal against *C. difficile*, and is also particularly notable for being very specific in its targeting of Clostridia, while not impacting upon other faecal bacteria^40^; this is in marked contrast to other anti-CDI agents. A recent study longitudinally profiled stool bile acids for patients treated for CDI either with vancomycin or ridinilazole, and compared the changes seen with the two agents.^41^ Over the course of treatment with vancomycin, an almost 100-fold increase in the ratio of conjugated to secondary bile acids was observed, consistent with other studies demonstrating that vancomycin use is associated with loss of faecal secondary bile acids and enrichment in primary unconjugated bile acids (likely reflecting its relatively broad anti-microbial actions).^42,43^ However, in comparison, only very modest changes from baseline were seen in the same bile acid profiles of ridinilazole-treated patients.^41^ The stool bile acid ratios seen at the end of treatment were shown to be predictive of which patients experienced future recurrence. As such, this experience with ridinilazole has helped to demonstrate that, in the continued hunt for novel anti-CDI antimicrobials, it is not only potency of action against the life cycle of *C. difficile* that is important to consider, but also the potential impact upon the bile-metabolising functionality of gut commensal bacteria.

### Faecal microbiota transplantation

FMT is now well-established as a safe and highly effective treatment option for patients with recurrent or refractory CDI.^44^ However, whilst it is recognised that FMT acts in such patients to restore the stool microbiome to a composition comparable with the pre-morbid state,^45–52^ the specific mechanisms underlying its efficacy have until recently remained poorly defined. The demonstration that either a defined consortium of commensal bacteria or spores derived from healthy donor stool deliver efficacy comparable with that of conventional FMT in treating rCDI support the concept that the bacterial component of FMT is a key contributor to efficacy.^53–55^ Of further particular interest has been the demonstration that sterile filtered FMT also is efficacious for treating rCDI.^56^ Collectively, these data suggest that soluble factors related to bacteria – but not necessarily intact bacteria *per se –* are important mediators of the efficacy of FMT, with potential explanations including bacterial products (e.g., enzymes or other proteins), associated bacteriophages, or gut microbial metabolites.^57^ Given these data described above demonstrating that a contributory factor to the pathogenesis of CDI is loss of gut microbiome members with bile-metabolising functionality, one recent area of focus has been as to whether one of the key mechanisms of FMT may be restoration of bacteria that produce these enzymes, and associated reversal of the abnormal bile acid milieu of the distal gut.

Consistent with this hypothesis, healthy donor stool typically has very low levels of TCA, but relatively high levels of secondary bile acids.^27,32,46,58–60^ Conversely, in human patients with rCDI, stool TCA is found at considerably increased levels compared with healthy donors, whilst secondary bile acids predominate in post-FMT stool.^27,32,46,58,59,61^ Exposure of *C. difficile* spores to the bile acid milieu found in antibiotic-treated mouse caecum or human stool pre-FMT was sufficient to cause spore germination,^31,62^ whereas that of the non-antibiotic-treated mouse caecum or human stool post-FMT prevented germination and vegetative growth of *C. difficile*.^31,62^ It has also been suggested that stool LCA may have utility as a predictor of FMT response, with sensitivity and specificity of >90%.^60^ Further experimental work has explored the impact of FMT upon microbial bile acid-metabolizing functionality in both rodent models and patients with rCDI.^27,63,64^ Patients with rCDI had a markedly reduced relative abundance of a broad range of BSH-producing bacteria within their stool microbiome pre-FMT in comparison with post-FMT, as well as in comparison with healthy stool donors.^27^ Successful FMT for rCDI rapidly and sustainably restored stool *bsh* gene copy number and BSH functionality from the very low levels detected pre-FMT up to high levels comparable with those of healthy stool donors,^27^ together with at least partial recovery of the *bai*CD operon of 7-α-dehydroxylase.^27^ Finally, stool *C. difficile* counts were reduced by approximately 70% in an rCDI mouse model after administration of *Escherichia coli* engineered to express highly active BSH compared with mice administered BSH-negative *E. coli*.^27^ Supportive of these data, in a metagenomic study of stool studies collected pre- and post-FMT for rCDI, analysis of gene functions demonstrated that secondary bile acid biosynthesis was one of the pathways most significantly restored by FMT.^64^ In totality, these data support the hypothesis that FMT-driven restoration of gut microbial bile acid metabolism is an important mechanism contributing to the efficacy of FMT in treating rCDI.

Within the field of FMT, much recent interest has been focussed on whether ‘microbiome therapeutics’ derived from commensal bacteria within healthy donor stool may have a role as a ‘next generation’ FMT product. While one such product – SER109, consisting of donor-derived purified spores – failed to meet its primary endpoint of CDI remission in a phase II study (potentially due to underdosing), it was observed that where early engraftment of the product occurred, this was associated with future non-recurrence, as well as with increased faecal secondary bile acids.^65^ Initial results from a phase III study of SER-109 undertaken by Seres Therapeutics (the ECOSPOR III study) have been reported recently, with the product reaching the primary trial endpoint for reducing CDI recurrence.^66^ Furthermore, Finch Therapeutics has also reported positive initial outcome data from a phase II study of an investigational ‘whole microbiome’ product (CP101; the PRISM3 trial).^67^ As such products emerge further within trials, their impact upon microbiota-mediated bile acid metabolism is likely to be a useful area of investigation.

## Other potential mechanisms by which gut microbiome–bile acid interactions may impact upon CDI

### Fibroblast growth factor–farnesoid X receptor pathway

One key mechanism through which bile acids communicate with the host is through their role as endogenous ligands for host cell receptors. These include the nuclear receptor farnesoid X receptor (FXR), and the G protein-coupled plasma membrane bile acid receptor TGR5, both of which exhibit varying affinities for different bile acids and their moieties.^16^ In humans, the most potent endogenous ligand for FXR is CDCA; DCA and LCA are moderate FXR agonists, whilst CA also has modest agonist activity.^16^ A direct link between microbiota, bile acids and FXR signalling has been demonstrated in rodents through the use of germ-free or antibiotic-treated animals.^16^ Furthermore, not only does the gut microbiota influence bile acid metabolism, but bile acids directly influence the survival and growth of gut microbiota constituents, including *via* FXR-mediated signalling. More specifically, in a study where healthy volunteers or mice were administered obeticholic acid (OCA; a bile acid analogue and FXR agonist), endogenous bile acid synthesis was suppressed, and a reversible induction of Gram-positive bacteria (particularly a relative enrichment in *Firmicutes*) was observed.^68^

A number of rodent and human studies have now established an association between perturbation of the FXR pathway and CDI. In a CDI mouse model, treatment with ursodeoxycholic acid (UDCA) was associated with increased transcripts related to FXR and fibroblast growth factor (FGF)-related signalling, as well as with reduced intestinal inflammation (Figure 1).^69^ Similarly, in patients with rCDI, successful FMT was associated with increased circulating FGF-19, again consistent with upregulated FXR activation.^70^ While the specific mechanistic underpinning this post-FMT increase in FXR activity is unclear, one theory was that the reduced level of a potent FXR agonist (CDCA) is offset by increased levels of two moderate FXR agonists (DCA and LCA), with a net upregulation of the ileal FXR–FGF pathway.^70,71^ A further interesting observation has been that successful FMT for rCDI is associated with a rapid and sustained reduction in the amount of tauro-β-muricholic acid in the stool of rCDI patients to a level comparable with that of healthy donors.^72^ Tauro-β-muricholic acid is of particular interest because this bile acid is a potent FXR antagonist (at least in mice, where levels are much higher than in humans), and therefore hypothesised to be a key intermediary contributing to the association between the gut microbiota and FXR signalling.^73^

A number of potential explanations have been proposed as to how upregulation of FXR signalling may contribute towards the resolution of CDI. Ileal FXR activation will result in reduction of further hepatic bile acid synthesis by negative feedback; this will result in the reduced further secretion of conjugated primary bile acids (including TCA) into bile and, therefore, into the small intestine, helping to limit further germination of *C. difficile*. Significantly increased hepatic primary bile acid production was observed in a high-fat diet CDI mouse model, and this was reduced towards normal levels through administration of OCA.^74^ OCA administration in this same study not only resulted in a reduced *C. difficile* burden, but also less diarrhoea and reduced intestinal inflammation.^74^ In addition, in a non-CDI/chemically induced rodent colitis model, OCA administration was again associated with reduced colonic inflammation as well as a more intact intestinal barrier,^75^ further suggesting that FXR may provide benefits beyond direct effects upon *C. difficile per se*. Of particular relevance to the setting of CDI, FXR activation has also been demonstrated to inhibit bacterial overgrowth in mouse ileum,^76^ and is associated with reduced expression of nuclear factor kappa B (NF-κB) target genes [including tumour necrosis factor alpha (TNF-α) and interleukin (IL)-1β] that regulate the host innate immune response.^77^ FXR agonists (including OCA in particular) have recently become of increasing clinical focus for their potential role in the treatment of gastrointestinal (GI) and liver diseases, and a case for CDI being one such condition of interest may clearly be made; however, at present, there have been no human trial data presented on the use of OCA as treatment for CDI.

### Other direct actions of bile acids

Gut microbiome–bile acid metabolism interactions also play a contributory role in the pathogenesis of even certain non-*C. difficile* diarrhoeal diseases. For instance, bile acids are recognised to directly impact significantly upon GI motility (reducing small intestinal transit time but increasing colonic transit time)^78,79^; furthermore, increased luminal bile acid levels result in increased chloride secretion and reduced fluid absorption, which may result in diarrhoea.^80^ These effects of bile acids are at least partly mediated by binding to specific host receptors; for instance, activation of the membrane Takeda G protein-coupled receptor (TGR-5; for which secondary bile acids are the major endogenous agonists) has an overall impact of increased colonic motility and reduced GI inflammation.^81,82^ Furthermore, in patients with chronic functional diarrhoea and/or bile acid diarrhoea, the proportion/percentage of stool primary bile acids is increased, and these values predict increased colonic transit.^83^ A proportion of such patients also demonstrate an increase in bile-metabolising *Clostridia* within their stool microbiome, increased faecal bile acid excretion, and reduced serum FGF-19.^84^ Increased intestinal transit time appears to result in a reduction in the extent to which microbially related bile acid biotransformations occur.^85^ However, the degree to which these processes may also contribute to the diarrhoea that typifies CDI are not presently well-defined.

### Enterohepatic circulation

An additional area of potential relevance relates to the enterohepatic circulation, that is, the ~95% of bile acids that are reabsorbed from the distal small gut. It is conjugated bile acids that are particularly absorbed, and the process is mediated principally *via* the apical sodium dependent bile acid transporter (ASBT) in the distal ileum. The ileum of germ-free rodents has increased capacity for the absorption of TCA compared with wild-type animals, while BSH-mediated deconjugation of glycine or taurine is associated with reduced active uptake of bile acids from the small intestine *via* ASBT.^16^ Given the apparent interaction between the gut microbiome and ASBT function – coupled with the key contribution of gut microbiome perturbation to CDI pathogenesis – it is clearly feasible that altered enterohepatic circulation may occur in CDI, although this has not been demonstrated experimentally. A further implication of this interaction relates to different bile acid patterns found in samples derived from the ileum to those from the colon of either patients or animal models of CDI; this may impact upon experimental design and data interpretation in future studies.

## Conclusions

The data presented here highlight the rapid advancements that have been made in the understanding of gut microbiome–bile-metabolism–FXR interactions, their relevance to the pathogenesis of CDI and their implications for therapeutic intervention in rCDI. However, despite such progress, there remain many ongoing areas of uncertainty and complexity where further research is needed. For instance, production by the gut microbiome of a particular secondary bile acid, 3β-hydrodeoxycholic acid, has been demonstrated recently to act *via* FXR to promote the generation of peripheral regulatory T cells,^86^ but the potential implications of this and other bile acid-immune interactions for CDI (and/or other gut inflammatory disorders) are currently of unclear significance. The specific pathways of synthesis and biotransformation (either host or microbial) of a large number of bile acids commonly detectable in biofluids remains poorly defined, with their potential contribution to disease states therefore remaining currently underexplored. Differences in the pool of bile acid species detected in rodents and humans introduces complexity in, for example, extrapolating results of mouse models to the interpretation of human disease states.

Furthermore, despite the growing body of work within this area, no current laboratory test of clinical applicability has been derived. For instance, there currently exists no stool or serum assay that assesses a microbiome/bile acid ‘signature’ – or specific concentrations/proportions of bile moieties from patients – to help with the assessment and management of CDI. A focus of future work may include aiming to develop such assays, for example, for the stratification/prediction of recurrent disease in patients with primary CDI or for the prediction of the requirement of further FMT in rCDI patients after a first administration. In addition, data thus far also suggest that manipulation of the gut microbiome–bile acid axis is also likely to remain an important area of focus as novel CDI therapeutics are developed.

## References

[1] Guh AY Mu Y Winston LG , et al. Trends in U.S. burden of *Clostridioides difficile* infection and outcomes. N Engl J Med 2020; 382: 1320–1330. 32242357 10.1056/NEJMoa1910215 PMC7861882

[2] Magill SS Edwards JR Bamberg W , et al. Multistate point-prevalence survey of health care–associated infections. N Engl J Med 2014; 370: 1198–1208. 24670166 10.1056/NEJMoa1306801 PMC4648343

[3] Lessa FC Mu Y Bamberg WM , et al. Burden of *Clostridium difficile* infection in the United States. N Engl J Med 2015; 372: 825–834. 25714160 10.1056/NEJMoa1408913 PMC10966662

[4] Khanna S Pardi DS Aronson SL , et al. The epidemiology of community-acquired *Clostridium difficile* infection: a population-based study. Am J Gastroenterol 2012; 107: 89–95. 22108454 10.1038/ajg.2011.398 PMC3273904

[5] Feuerstadt P Das R Brandt LJ. The evolution of urban *C. difficile* infection (CDI): CDI in 2009–2011 is less severe and has better outcomes than CDI in 2006–2008. Am J Gastroenterol 2014; 109: 1265–1276. 25001255 10.1038/ajg.2014.167

[6] Leffler DA Lamont JT. *Clostridium difficile* infection. N Engl J Med 2015; 372: 1539–1548. 25875259 10.1056/NEJMra1403772

[7] Thomas C Stevenson M Riley TV. Antibiotics and hospital-acquired *Clostridium difficile*-associated diarrhoea: a systematic review. J Antimicrob Chemother 2003; 51: 1339–1350. 12746372 10.1093/jac/dkg254

[8] Anand A Glatt AE. *Clostridium difficile* infection associated with antineoplastic chemotherapy: a review. Clin Infect Dis 1993; 17: 109–113. 8353229 10.1093/clinids/17.1.109

[9] Bourgault A-M Lamothe F Toye B , et al. Host and pathogen factors for *Clostridium difficile* infection and colonization. N Engl J Med 2011; 365: 1693–1703. 22047560 10.1056/NEJMoa1012413

[10] Howell MD Novack V Grgurich P , et al. Iatrogenic gastric acid suppression and the risk of nosocomial *Clostridium difficile* infection. Arch Intern Med 2010; 170: 784–790. 20458086 10.1001/archinternmed.2010.89

[11] Bliss DZ Johnson S Savik K , et al. Acquisition of *Clostridium difficile* and *Clostridium difficile*-associated diarrhea in hospitalized patients receiving tube feeding. Ann Intern Med 1998; 129: 1012–1019. 9867755 10.7326/0003-4819-129-12-199812150-00004

[12] Martin JSH Monaghan TM Wilcox MH. *Clostridium difficile* infection: epidemiology, diagnosis and understanding transmission. Nat Rev Gastroenterol Hepatol 2016; 13: 206–216. 26956066 10.1038/nrgastro.2016.25

[13] Allegretti JR Mullish BH Kelly C , et al. The evolution of the use of faecal microbiota transplantation and emerging therapeutic indications. Lancet 2019; 394: 420–431. 31379333 10.1016/S0140-6736(19)31266-8

[14] Holmes E Li JV Marchesi JR , et al. Gut microbiota composition and activity in relation to host metabolic phenotype and disease risk. Cell Metabolism 2012; 16: 559–564. 23140640 10.1016/j.cmet.2012.10.007

[15] Krautkramer KA Fan J Bäckhed F. Gut microbial metabolites as multi-kingdom intermediates. Nat Rev Microbiol 2021; 19: 77–94. 32968241 10.1038/s41579-020-0438-4

[16] Wahlström A Sayin SI Marschall HU , et al. Intestinal crosstalk between bile acids and microbiota and its impact on host metabolism. Cell Metab 2016; 24: 41–50. 27320064 10.1016/j.cmet.2016.05.005

[17] Mullish BH Pechlivanis A Barker GF , et al. Functional microbiomics: evaluation of gut microbiota-bile acid metabolism interactions in health and disease. Methods. Epub ahead of print 26 April 2018. DOI: 10.1016/j.ymeth.2018.04.028. PMC6347095 29704662

[18] Ridlon JM Kang D-J Hylemon PB. Bile salt biotransformations by human intestinal bacteria. J Lipid Res 2006; 47: 241–259. 16299351 10.1194/jlr.R500013-JLR200

[19] Jones BV Begley M Hill C , et al. Functional and comparative metagenomic analysis of bile salt hydrolase activity in the human gut microbiome. Proc Natl Acad Sci U S A 2008; 105: 13580–13585. 18757757 10.1073/pnas.0804437105 PMC2533232

[20] Song Z Cai Y Lao X , et al. Taxonomic profiling and populational patterns of bacterial Bile Salt Hydrolase (BSH) genes based on worldwide human gut microbiome. Microbiome 2019; 7: 9. 30674356 10.1186/s40168-019-0628-3 PMC6345003

[21] Kitahara M Takamine F Imamura T , et al. Assignment of Eubacterium sp. VPI 12708 and related strains with high bile acid 7alpha-dehydroxylating activity to *Clostridium* scindens and proposal of *Clostridium* hylemonae sp. nov., isolated from human faeces. Int J Syst Evol Microbiol 2000; 50: 971–978. 10843034 10.1099/00207713-50-3-971

[22] Sorg JA Sonenshein AL. Bile salts and glycine as cogerminants for *Clostridium difficile* spores. J Bacteriol 2008; 190: 2505–2512. 18245298 10.1128/JB.01765-07 PMC2293200

[23] Thanissery R Winston JA Theriot CM. Inhibition of spore germination, growth, and toxin activity of clinically relevant *C. difficile* strains by gut microbiota derived secondary bile acids. Anaerobe 2017; 45: 86–100. 28279860 10.1016/j.anaerobe.2017.03.004 PMC5466893

[24] Theriot CM Bowman AA Young VB. Antibiotic-induced alterations of the gut microbiota alter secondary bile acid production and allow for *Clostridium difficile* spore germination and outgrowth in the large intestine. mSphere 2016; 1: e00045-15. 10.1128/mSphere.00045-15 PMC4863611 27239562

[25] Tam J Icho S Utama E , et al. Intestinal bile acids directly modulate the structure and function of *C. difficile* TcdB toxin. Proc Natl Acad Sci U S A 2020; 117: 6792–6800. 32152097 10.1073/pnas.1916965117 PMC7104382

[26] Sorg JA Sonenshein AL. Chenodeoxycholate is an inhibitor of *Clostridium difficile* spore germination. J Bacteriol 2009; 191: 1115–1117. 19060152 10.1128/JB.01260-08 PMC2632082

[27] Mullish BH McDonald JAK Pechlivanis A , et al. Microbial bile salt hydrolases mediate the efficacy of faecal microbiota transplant in the treatment of recurrent *Clostridioides difficile* infection. Gut 2019; 68: 1791–1800. 30816855 10.1136/gutjnl-2018-317842 PMC6839797

[28] Sorg JA Sonenshein AL. Inhibiting the initiation of *Clostridium difficile* spore germination using analogs of chenodeoxycholic acid, a bile acid. J Bacteriol 2010; 192: 4983–4990. 20675492 10.1128/JB.00610-10 PMC2944524

[29] Francis MB Allen CA Shrestha R , et al. Bile acid recognition by the *Clostridium difficile* germinant receptor, CspC, is important for establishing infection. PLoS Pathog 2013; 9: e1003356. 10.1371/journal.ppat.1003356 PMC3649964 23675301

[30] Mekhjian HS Phillips SF Hofmann AF. Colonic absorption of unconjugated bile acids - perfusion studies in man. Dig Dis Sci 1979; 24: 545–550. 456241 10.1007/BF01489324

[31] Theriot CM Koenigsknecht MJ Carlson PE , et al. Antibiotic-induced shifts in the mouse gut microbiome and metabolome increase susceptibility to *Clostridium difficile* infection. Nat Commun 2014; 5: 3114. 24445449 10.1038/ncomms4114 PMC3950275

[32] Allegretti JR Kearney S Li N , et al. Recurrent *Clostridium difficile* infection associates with distinct bile acid and microbiome profiles. Aliment Pharmacol Ther 2016; 43: 1142–1153. 27086647 10.1111/apt.13616 PMC5214573

[33] Buffie CG Bucci V Stein RR , et al. Precision microbiome reconstitution restores bile acid mediated resistance to *Clostridium difficile*. Nature 2014; 517: 205–208. 25337874 10.1038/nature13828 PMC4354891

[34] Studer N Desharnais L Beutler M , et al. Functional intestinal bile acid 7α-dehydroxylation by *Clostridium* scindens associated with protection from *Clostridium difficile* infection in a gnotobiotic mouse model. Front Cell Infect Microbiol 2016; 6: 191. 28066726 10.3389/fcimb.2016.00191 PMC5168579

[35] Kang JD Myers CJ Harris SC , et al. Bile acid 7α-dehydroxylating gut bacteria secrete antibiotics that inhibit *Clostridium difficile*: role of secondary bile acids. Cell Chem Biol 2019; 26: 27–34.e4. 10.1016/j.chembiol.2018.10.003 PMC6338514 30482679

[36] Allegretti JR Mullish BH Bogart E , et al. 25 - microbiome and metabolic markers of *Clostridium difficile* recurrance. Gastroenterology 2018; 154: S-8–S-9.

[37] McDonald LC Gerding DN Johnson S , et al. Clinical practice guidelines for *Clostridium difficile* infection in adults and children: 2017 update by the Infectious Diseases Society of America (IDSA) and Society for Healthcare Epidemiology of America (SHEA). Clin Infect Dis 2018; 31: 431–455. 10.1093/cid/cix1085 PMC6018983 29462280

[38] Vickers RJ Tillotson GS Nathan R , et al. Efficacy and safety of ridinilazole compared with vancomycin for the treatment of *Clostridium difficile* infection: a phase 2, randomised, double-blind, active-controlled, non-inferiority study. Lancet Infect Dis 2017; 17: 735–744. 28461207 10.1016/S1473-3099(17)30235-9 PMC5483507

[39] Carlson TJ Endres BT Bassères E , et al. Ridinilazole for the treatment of *Clostridioides difficile* infection. Expert Opin Investig Drugs 2019; 28: 303–310. 10.1080/13543784.2019.1582640 30767587

[40] Vickers RJ Tillotson G Goldstein EJC , et al. Ridinilazole: a novel therapy for *Clostridium difficile* infection. Int J Antimicrob Agents 2016; 48: 137–143. 27283730 10.1016/j.ijantimicag.2016.04.026

[41] Qian X Yanagi K Kane AV , et al. Ridinilazole, a narrow spectrum antibiotic for treatment of *Clostridioides difficile* infection, enhances preservation of microbiota-dependent bile acids. Am J Physiol Liver Physiol 2020; 319: G227–G237. 10.1152/ajpgi.00046.2020 PMC7500266 32597706

[42] Vrieze A Out C Fuentes S , et al. Impact of oral vancomycin on gut microbiota, bile acid metabolism, and insulin sensitivity. J Hepatol 2014; 60: 824–831. 24316517 10.1016/j.jhep.2013.11.034

[43] Reijnders D Goossens GH Hermes GDA , et al. Clinical and translational report effects of gut microbiota manipulation by antibiotics on host metabolism in obese humans: a randomized double-blind placebo-controlled trial cell metabolism clinical and translational report effects of gut microbiota mani. Cell Metab 2016; 24: 63–74. 27411009 10.1016/j.cmet.2016.06.016

[44] Baunwall SMD Lee MM Eriksen MK , et al. Faecal microbiota transplantation for recurrent *Clostridioides difficile* infection: an updated systematic review and meta-analysis. EClinicalMedicine. Epub ahead of print 1 December 2020. DOI: 10.1016/j.eclinm.2020.100642. PMC7788438 33437951

[45] Weingarden A González A Vázquez-Baeza Y , et al. Dynamic changes in short- and long-term bacterial composition following fecal microbiota transplantation for recurrent *Clostridium difficile* infection. Microbiome 2015; 3: 10. 25825673 10.1186/s40168-015-0070-0 PMC4378022

[46] Weingarden AR Chen C Bobr A , et al. Microbiota transplantation restores normal fecal bile acid composition in recurrent *Clostridium difficile* infection. AJP Gastrointest Liver Physiol 2014; 306: G310–G319. 10.1152/ajpgi.00282.2013 PMC3920123 24284963

[47] Hamilton MJ Weingarden AR Unno T , et al. High-throughput DNA sequence analysis reveals stable engraftment of gut microbiota following transplantation of previously frozen fecal bacteria. Gut Microbes 2013; 4: 125–135. 23333862 10.4161/gmic.23571 PMC3595072

[48] Staley C Vaughn BP Graiziger CT , et al. Community dynamics drive punctuated engraftment of the fecal microbiome following transplantation using freeze-dried, encapsulated fecal microbiota. Gut Microbes 2017; 8: 276–288. 28282270 10.1080/19490976.2017.1299310 PMC5479395

[49] Staley C Kaiser T Vaugh BP , et al. Predicting recurrence of *Clostridium difficile* infection following encapsulated fecal microbiota transplantation. Microbiome 2018; 6: 166. 30227892 10.1186/s40168-018-0549-6 PMC6145197

[50] Shankar V Hamilton MJ Khoruts A , et al. Species and genus level resolution analysis of gut microbiota in *Clostridium difficile* patients following fecal microbiota transplantation. Microbiome 2014; 2: 13. 24855561 10.1186/2049-2618-2-13 PMC4030581

[51] Smillie CS Sauk J Gevers D , et al. Strain tracking reveals the determinants of bacterial engraftment in the human gut following fecal microbiota transplantation. Cell Host Microbe 2018; 23: 229–240.e5. 10.1016/j.chom.2018.01.003 PMC8318347 29447696

[52] Kao D Roach B Silva M , et al. Effect of oral capsule– vs colonoscopy-delivered fecal microbiota transplantation on recurrent *Clostridium difficile* infection: a randomized clinical trial. JAMA 2017; 318: 1985–1993. 29183074 10.1001/jama.2017.17077 PMC5820695

[53] Petrof EO Gloor GB Vanner SJ , et al. Stool substitute transplant therapy for the eradication of *Clostridium difficile* infection: ‘RePOOPulating’ the gut. Microbiome 2013; 1: 3. 24467987 10.1186/2049-2618-1-3 PMC3869191

[54] Kao D Wong K Franz R , et al. The effect of a microbial ecosystem therapeutic (MET-2) on recurrent *Clostridioides difficile* infection: a phase 1, open-label, single-group trial. Lancet Gastroenterol Hepatol. Epub ahead of print 22 February 2021. DOI: 10.1016/S2468-1253(21)00007-8. 33631102

[55] Khanna S Pardi DS Kelly CR , et al. A novel microbiome therapeutic increases gut microbial diversity and prevents recurrent *Clostridium difficile* infection. J Infect Dis 2016; 214: 173–181. 26908752 10.1093/infdis/jiv766

[56] Ott SJ Waetzig GH Rehman A , et al. Efficacy of sterile fecal filtrate transfer for treating patients with *Clostridium difficile* infection. Gastroenterology 2017; 152: 799–811.e7. 10.1053/j.gastro.2016.11.010 27866880

[57] Segal JP Mullish BH Quraishi MN , et al. Mechanisms underpinning the efficacy of faecal microbiota transplantation in treating gastrointestinal disease. Therap Adv Gastroenterol 2020; 13: 175628482094690. 10.1177/1756284820946904 PMC7475788 32952613

[58] Staley C Weingarden AR Khoruts A , et al. Interaction of gut microbiota with bile acid metabolism and its influence on disease states. Appl Microbiol Biotechnol 2017; 101: 47–64. 27888332 10.1007/s00253-016-8006-6 PMC5203956

[59] Brown JR-M Flemer B Joyce SA , et al. Changes in microbiota composition, bile and fatty acid metabolism, in successful faecal microbiota transplantation for *Clostridioides difficile* infection. BMC Gastroenterol 2018; 18: 131. 30153805 10.1186/s12876-018-0860-5 PMC6114236

[60] Farowski F Solbach P Tsakmaklis A , et al. Potential biomarkers to predict outcome of faecal microbiota transfer for recurrent *Clostridioides difficile* infection. Dig Liver Dis 2019; 51: 944–951. 30770201 10.1016/j.dld.2019.01.012

[61] Seekatz AM Theriot CM Rao K , et al. Restoration of short chain fatty acid and bile acid metabolism following fecal microbiota transplantation in patients with recurrent *Clostridium difficile* infection. Anaerobe 2018; 53: 64–73. 29654837 10.1016/j.anaerobe.2018.04.001 PMC6185828

[62] Weingarden AR Dosa PI DeWinter E , et al. Changes in colonic bile acid composition following fecal microbiota transplantation are sufficient to control *Clostridium difficile* germination and growth. PLoS One 2016; 11: e0147210. 10.1371/journal.pone.0147210 PMC4720481 26789728

[63] Littmann ER Lee J-J Denny JE , et al. Host immunity modulates the efficacy of microbiota transplantation for treatment of *Clostridioides difficile* infection. Nat Commun 2021; 12: 1–15. 33531483 10.1038/s41467-020-20793-x PMC7854624

[64] Fujimoto K Kimura Y Allegretti JR , et al. Functional restoration of bacteriomes and viromes by fecal microbiota transplantation. Gastroenterology. Epub ahead of print 9 February 2021. DOI: 10.1053/j.gastro.2021.02.013. PMC8684800 33577875

[65] McGovern BH Ford CB Henn MR , et al. SER-109, an investigational microbiome drug to reduce recurrence after *Clostridioides difficile* infection: lessons learned from a phase 2 trial. Clin Infect Dis. Epub ahead of print 7 April 2020. DOI: 10.1093/cid/ciaa387. PMC8204772 32255488

[66] Seres Therapeutics. Seres therapeutics announces positive topline results from SER-109 phase 3 ECOSPOR III study in recurrent *C. difficile Infection*, https://ir.serestherapeutics.com/news-releases/news-release-details/seres-therapeutics-announces-positive-topline-results-ser-109 (accessed 9 February 2021).

[67] Business Wire. Finch therapeutics announces positive topline results from randomized controlled trial of CP101, an oral microbiome drug, for the prevention of recurrent *C. difficile* infection, https://www.businesswire.com/news/home/20200619005011/en/Finch-Therapeutics-Announces-Positive-Topline-Results-from-Randomized-Controlled-Trial-of-CP101-an-Oral-Microbiome-Drug-for-the-Prevention-of-Recurrent-C.-difficile-Infection (accessed 9 February 2021).

[68] Friedman ES Li Y Shen TCD , et al. FXR-dependent modulation of the human small intestinal microbiome by the bile acid derivative obeticholic acid. Gastroenterology 2018; 155: 1741–1752.e5. 30144429 10.1053/j.gastro.2018.08.022 PMC6279623

[69] Winston JA Rivera AJ Cai J , et al. Ursodeoxycholic acid (udca) mitigates the host inflammatory response during *Clostridioides difficile* infection by altering gut bile acids. Infect Immun. Epub ahead of print 1 June 2020. DOI: 10.1128/IAI.00045-20. PMC7240095 32205405

[70] Monaghan T Mullish BH Patterson J , et al. Effective fecal microbiota transplantation for recurrent *Clostridioides difficile* infection in humans is associated with increased signalling in the bile acid-farnesoid X receptor-fibroblast growth factor pathway. Gut Microbes 2019; 10: 1–7. 30183484 10.1080/19490976.2018.1506667 PMC6546339

[71] Parks DJ Blanchard SG Bledsoe RK , et al. Bile acids: natural ligands for an orphan nuclear receptor. Science 1999; 284: 1365–1368. 10334993 10.1126/science.284.5418.1365

[72] Martinez-Gili L McDonald JAK Liu Z , et al. Understanding the mechanisms of efficacy of fecal microbiota transplant in treating recurrent *Clostridioides difficile* infection and beyond: the contribution of gut microbial-derived metabolites. Gut Microbes 2020; 12: 1810531. 32893721 10.1080/19490976.2020.1810531 PMC7524310

[73] Sayin SI Wahlström A Felin J , et al. Gut microbiota regulates bile acid metabolism by reducing the levels of tauro-beta-muricholic acid, a naturally occurring FXR antagonist. Cell Metab 2013; 17: 225–235. 23395169 10.1016/j.cmet.2013.01.003

[74] Jose S Mukherjee A Horrigan O , et al. Obeticholic acid ameliorates severity of *Clostridioides difficile* infection in high fat diet-induced obese mice. Mucosal Immunol. Epub ahead of print 18 August 2020. DOI: 10.1038/s41385-020-00338-7. PMC7889747 32811993

[75] Gadaleta RM van Erpecum KJ Oldenburg B , et al. Farnesoid X receptor activation inhibits inflammation and preserves the intestinal barrier in inflammatory bowel disease. Gut 2011; 60: 463–472. 21242261 10.1136/gut.2010.212159

[76] Inagaki T Moschetta A Lee Y-K , et al. Regulation of antibacterial defense in the small intestine by the nuclear bile acid receptor. Proc Natl Acad Sci U S A 2006; 103: 3920–3925. 16473946 10.1073/pnas.0509592103 PMC1450165

[77] Vavassori P Mencarelli A Renga B , et al. The bile acid receptor FXR is a modulator of intestinal innate immunity. J Immunol 2009; 183: 6251–6261. 19864602 10.4049/jimmunol.0803978

[78] Armstrong DN Krenz HK Modlin IM , et al. Bile salt inhibition of motility in the isolated perfused rabbit terminal ileum. Gut 1993; 34: 483–488. 8491394 10.1136/gut.34.4.483 PMC1374307

[79] Shiff SJ Soloway RD Snape WJ. Mechanism of deoxycholic acid stimulation of the rabbit colon. J Clin Invest 1982; 69: 985–992. 7076855 10.1172/JCI110538 PMC370153

[80] Keely SJ Walters JRF . The farnesoid X receptor: good for BAD. CMGH 2016; 2: 725–732. 28174746 10.1016/j.jcmgh.2016.08.004 PMC5247348

[81] Alemi F Poole DP Chiu J , et al. The receptor TGR5 mediates the prokinetic actions of intestinal bile acids and is required for normal defecation in mice. Gastroenterology 2013; 144: 145–154. 23041323 10.1053/j.gastro.2012.09.055 PMC6054127

[82] Cipriani S Mencarelli A Chini MG , et al. The bile acid receptor GPBAR-1 (TGR5) modulates integrity of intestinal barrier and immune response to experimental colitis. PLoS One 2011; 6: e25637. 10.1371/journal.pone.0025637 PMC3203117 22046243

[83] Vijayvargiya P Camilleri M Chedid V , et al. Analysis of fecal primary bile acids detects increased stool weight and colonic transit in patients with chronic functional diarrhea. Clin Gastroenterol Hepatol 2019; 17: 922–929.e2. 29902647 10.1016/j.cgh.2018.05.050 PMC6291372

[84] Zhao L Yang W Chen Y , et al. A Clostridia-rich microbiota enhances bile acid excretion in diarrhea-predominant irritable bowel syndrome. J Clin Invest 2020; 130: 438–450. 31815740 10.1172/JCI130976 PMC6934182

[85] Marcus SN Heaton KW. Liver and biliary intestinal transit, deoxycholic acid and the cholesterol saturation of bile-three inter-related factors. Gut 1986; 27: 550–558. 3699564 10.1136/gut.27.5.550 PMC1433498

[86] Campbell C McKenney PT Konstantinovsky D , et al. Bacterial metabolism of bile acids promotes generation of peripheral regulatory T cells. Nature 2020; 581: 475–479. 32461639 10.1038/s41586-020-2193-0 PMC7540721

